# Individual Characteristics Associated with PBDE Levels in U.S. Human Milk Samples

**DOI:** 10.1289/ehp.0900759

**Published:** 2009-09-14

**Authors:** Julie L. Daniels, I-Jen Pan, Richard Jones, Sarah Anderson, Donald G. Patterson, Larry L. Needham, Andreas Sjödin

**Affiliations:** 1 Department of Epidemiology and; 2 Department of Maternal and Child Health, University of North Carolina School of Public Health, Chapel Hill, North Carolina, USA;; 3 Organic Analytic Toxicology Branch, National Center for Environmental Health, Centers for Disease Control and Prevention, Atlanta, Georgia, USA;; 4 EnviroSolutions Consulting, Inc., Jasper, Georgia, USA

**Keywords:** brominated flame retardants, environment, epidemiology, human milk, infant, lactation, PBDE, persistent pollutants, polybrominated diphenyl ethers, pregnancy

## Abstract

**Background:**

Reported polybrominated diphenyl ether (PBDE) concentrations in human samples in the United States have been higher than in Europe and Asia. Little is known about factors that contribute to individual variability in body burden.

**Objective:**

In this large study we measured PBDE concentrations in human milk from the United States during 2004–2006. We assessed characteristics associated with concentrations in milk and change in milk concentration between 3 and 12 months postpartum.

**Methods:**

We analyzed 303 milk samples obtained 3 months postpartum for PBDEs. A second sample was analyzed for 83 women still lactating 12 months postpartum. PBDE concentrations in milk and variability by individual characteristics such as age, parity, and prepregnancy body mass index (BMI) were evaluated using generalized linear models.

**Results:**

PBDE congeners BDEs 28, 47, 99, 100, and 153 were detected in > 70% of samples. BDE-47 concentrations were the highest, ranging from below the limit of detection to 1,430 ng/g lipid, with a median of 28 ng/g lipid. Concentrations of most individual PBDE congeners and the sum of BDEs 28, 47, 99, 100, and 153 (∑PBDE) were lower among mothers > 34 years of age compared with those 25–29 years of age and higher among mothers with high compared with normal BMI, after adjustment for other covariates. Parity was not associated with PBDE concentration. The change in ∑PBDE concentration in milk between 3 and 12 months postpartum was highly variable (median increase, 14%; interquartile range, −26% to 50%).

**Conclusions:**

PBDEs were detected in nearly all human milk samples, varying by maternal weight and age and over the course of breast-feeding.

Polybrominated diphenyl ethers (PBDEs) are high-production-volume chemicals that have been widely used as flame retardants in electronic equipment, carpet, and the polyurethane foam used in furniture (Darnerud et al. 2001; Frederiksen et al. 2009; Sjodin et al. 2003). Unlike reactive flame retardants, which are mixed into plastic before polymerization to form a covalent bond with the polymer matrix, PBDEs are additive flame retardants that are mixed with the finished polymer. Additives have a greater potential to leach from the product into the environment over the life cycle of the product (Sjodin et al. 2003).

Commercial PBDE formulations are named by their average degree of bromination. The pentaBDE formulation, used in polyurethane foam in carpet padding and furniture, contains mainly tetra- through hexa-BDEs. The octa and deca formulations are used in the hard plastics of electronic casings and similar products. The octa formulation includes octa- and hepta-congeners, whereas decaBDE is almost entirely BDE-209. The formulations with 7–10 bromines have relatively short half-lives in humans compared with congeners with shorter bromine chains (Lorber 2008; Thuresson et al. 2006). The penta and octa formulations were banned in Europe at the turn of the century and voluntarily withdrawn in the United States in 2004 because of their persistence in the environment and unknown safety (Lind et al. 2003; Lorber 2008; Meironyte et al. 1999; Noren and Meironyte 2000). DecaBDE continues to be produced in North America and much of Europe (Darnerud et al. 2001; de Wit 2002; Hale et al. 2003; Sjodin 2004; Sjodin et al. 2003).

Detection of PBDEs in the environment and in human tissues has dramatically increased over the past 20 years due to high production and use as well as persistence in the environment (Darnerud et al. 2001; Frederiksen et al. 2009; Meironyte et al. 1999; Noren and Meironyte 2000; Petreas et al. 2003; Sjodin et al. 2004a). Levels in the United States and Canada more than doubled in the late 1990s and are estimated to be among the highest in the world (Betts 2002; Darnerud et al. 2001; Sjodin et al. 2003, 2004a, 2008a, 2008b; Zhu and Hites 2004).

The ubiquitous nature of these chemicals is demonstrated by measurable levels in samples of air, water, dust, and sewage sludge, but the paths by which humans are exposed to PBDEs are still unclear (Hale et al. 2002, 2003; Watanabe and Sakai 2003). Although diet may contribute to adult PBDE levels (Darnerud et al. 2001; Fraser et al. 2009), exposure to dust in the indoor environment may be a more significant contributor (Johnson-Restrepo and Kannan 2009; Sjodin et al. 2008a). This differs from traditional persistent organic pollutants, such as polychlorinated biphenyls (PCBs), for which diet is the main route of exposure (Frederiksen et al. 2009; Lorber 2008).

For infants, the greatest source of PBDE exposure is believed to be through their mothers, both *in utero* and from breast-feeding (Johnson-Restrepo and Kannan 2009; Toms et al. 2008). High PBDEs have been measured in the placenta, cord blood, and liver tissue (Costa and Giordano 2007; Doucet et al. 2009; Frederiksen et al. 2009; Schecter et al. 2007). Maternal and cord blood levels are highly correlated, as are maternal blood and breast milk levels (Guvenius et al. 2003; Mazdai et al. 2003). Because the compounds distribute in tissues based on the lipid concentration, the high lipid concentration in milk enhances exposure for the nursing infant (Darnerud et al. 2001).

The few studies measuring PBDEs in breast milk in the United States have occurred between 2002 and 2006 (Table 1) (Johnson-Restrepo et al. 2007; Schecter et al. 2003; She et al. 2007; Wu et al. 2007). Most of these studies have been small and provided little information about how PBDE levels varied among individuals or with duration of breast-feeding. We report PBDE concentrations in milk from a large sample of women in central North Carolina (USA). We evaluated the correlation between PBDEs and other persistent pollutants. We also assessed factors that might contribute to individual variability, including age, parity, and body mass index (BMI), and calculated the change in milk concentration over the first postpartum year.

## Materials and Methods

### Study population

Participants in this analysis were women who participated in the later years of the Pregnancy, Infection, and Nutrition (PIN) Study and PIN Postpartum Study. These two studies followed central North Carolina women from early pregnancy through 12 months postpartum between 2001 and 2005 (PIN 2009). Women were recruited into PIN from the University of North Carolina prenatal care clinics and delivered their infants at University of North Carolina hospitals (64% of eligible women participated during pregnancy). Seventy-three percent of eligible participants from the PIN Study (delivered a singleton infant without major birth defects) continued in the PIN Postpartum Study. In January 2004, the PIN Babies Study began collecting milk samples at 3 and 12 months postpartum from lactating women. Between 2004 and 2006, 331 of 589 eligible women were lactating, and so were eligible to contribute milk samples at 3 months postpartum.

A research team visited the women’s homes at 3 and 12 months postpartum to collect milk, take anthropometric measurements of the mother, assess her percent body fat using a bioelectrical impedance scale, and interview her about her diet, psychosocial health, physical activity, lifestyle, and breast-feeding practices. Before the scheduled visit, a milk collection kit was mailed to mothers who said they were breast-feeding. Women were instructed to empty both breasts by pumping at around 1000 hours on the morning of her scheduled home visit, gently mix the milk, use the plastic pipette to transfer milk into Eppendorf tubes, and freeze the milk until the staff arrived later that day. Milk samples were transported on ice and then stored at −80°C. All study protocols were approved by the Institutional Review Board at the University of North Carolina–Chapel Hill, and all participants provided informed consent.

Analysis of PBDEs, PCBs, and *p*,*p*′-DDE (dichlorodiphenyldichloroethylene) in milk was conducted at the Organic Analytic Toxicology Branch of the National Center for Environmental Health at the Centers for Disease Control and Prevention using previously described methodology (Sjodin et al. 2004b). Milk samples underwent solid-phase dispersion onto diatomaceous earth, and internal standards were added. Lipids and target analytes were extracted using a solid-phase extraction system, in which the sample on diatomaceous earth was dried by pressurized nitrogen and extracted with dichloromethane. Lipid content was determined gravimetrically, and the final analytical determination of PBDEs, PCBs, and *p*,*p*′-DDE was performed by gas chromatography/isotope-dilution high-resolution mass spectrometry. Two control and two blank samples were added to each batch of 16 unknown samples for quality control. Laboratory quality assurance practices were regularly monitored (Sjodin et al. 2004b).

### Data analysis

PBDEs in the milk samples provided at 3 months postpartum appeared log-normally distributed; thus medians, quartiles, and total range are reported here. The concentration of target analytes were lipid normalized and are reported as nanograms per gram of milk lipids. Samples below the limit of detection (LOD) were imputed three ways in the calculations: 0, nd. The method of including observations below the LOD did not affect the results; thus only results based on are presented here.

The congeners detected in more than 70% of the individuals’ samples (BDEs 28, 47, 99, 100, and 153) were summed to produce a cumulative measure (∑PBDE). Spearman correlation coefficients (*r*_S_) were calculated to assess the correlations among the PBDE congeners and among ∑PBDEs, ∑PCBs, and *p*,*p*′-DDE. For the women providing samples at 3 and 12 months postpartum, the correlation between the two samples was calculated, along with the percent change between 3 and 12 months: [(12-month concentration) – (3-month concentration)]/(3 month concentration) × 100%]. PBDE body burden (micrograms) was estimated as [concentration of PBDE (nanograms per gram lipid)] × [maternal body weight (kilograms)] × (body fat percentage at 3 or 12 months). Because the data were highly right-skewed, the sign test was used, instead of a paired *t-*test, to test the null hypothesis that the median change between 3 and 12 months postpartum equaled zero (α = 0.05).

We used linear models (PROC GLM, version 9.1; SAS Institute Inc., Cary, NC) to assess whether individual characteristics were associated with PBDE levels and to calculate adjusted geometric means for each category of each covariate, including maternal age (< 25, 25–29, 30–34, ≥ 35 years), parity (0, ≥ 1), race (nonwhite, white), and prepregnancy BMI (low, < 19.8 kg/m^2^; normal, 19.8 to ≤ 26.0 kg/m^2^; overweight, > 26.0 to ≤ 29.0 kg/m^2^; obese, > 29.0 kg/m^2^). Because PBDEs are stored in fat and prepregnancy BMI reflects a woman’s long-term fat stores, results are presented for prepregnancy BMI. However, we compared these results with two additional modeling approaches: *a*) adjusting for pregnancy weight gain and percent body fat at 3 months postpartum, and *b*) substituting prepregnancy BMI with 3-month postpartum BMI. The primary reason for not focusing on postpartum BMI measures is that interpreting the effects of postpartum BMI on long-term chemical storage medium is confounded by temporary fat and fluid gains associated with pregnancy.

## Results

Study participants were primarily college educated (82%) and white (86%). The mean maternal age was 30.7 ± 4.9 years, and 52% were primiparous. Three hundred four (62%) of the 334 lactating women provided a milk sample around 3 months postpartum (mean ± SD, 3.5 ± 0.6 months postpartum); PBDEs were successfully quantified in all but one. Eighty-three women (27%) also provided a milk sample at 12 months postpartum. Among congeners detected in > 70% of the samples, ∑PBDEs ranged from 1 to 2,010 ng/g lipid in the 3-month samples (Table 2). BDE-47 was present in highest concentration, which ranged from < LOD to 1,430 ng/g lipid. Among the congeners present in > 70% of samples, BDEs 28, 47, 99, and 100 were highly correlated with each other (*r*_S_ range, 0.8–0.9, *p* < 0.0001) and moderately correlated with BDE-153 (*r*_S_ range, 0.6–0.8, *p* < 0.0001; data not shown). ∑PBDE was not correlated with ∑PCBs (*r*_S_ = −0.1, *p* = 0.3) or *p*,*p*′-DDE (*r*_S_ = −0.1, *p* = 0.2) (data not shown).

**Figure 1:**
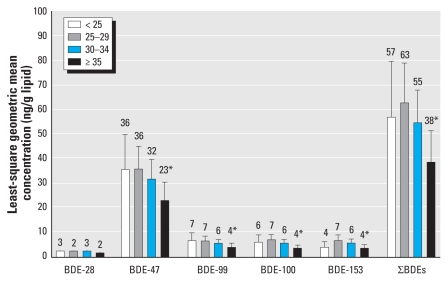
PBDE congener concentrations stratified by age (*x*-axis; years) and adjusted for parity, prepregnancy BMI, and race. Numbers above the bars are means; error bars indicate 95% confidence intervals. Sample sizes for each age group: 32 for < 25 years, 88 for 25–29 years, 130 for 30–34 years, and 54 for ≥ 35 years. *p < 0.05 in generalized linear model comparing with reference group (25–29 years).

**Figure 2:**
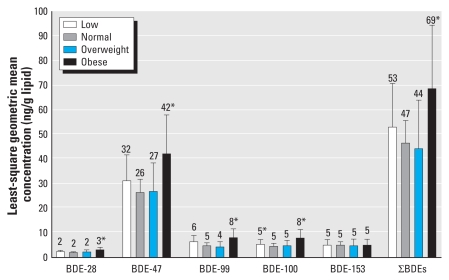
PBDE congener concentrations stratified by prepregnancy BMI (*x*-axis) and adjusted for age, parity, and race. Numbers above the bars are means; error bars indicate 95% confidence intervals. Sample sizes for each BMI group: 52 for low (< 19.8 kg/m^2^), 189 for normal (19.8 to ≤ 26.0 kg/m^2^), 27 for overweight (> 26.0 to ≤ 29.0 kg/m^2^), and 36 for obese (> 29.0 kg/m^2^). *p < 0.05 in generalized linear model compared with reference group (normal).

The milk concentrations of most individual PBDE congeners and ∑PBDE varied by maternal age and BMI. Mean concentrations were lower among women > 34 years of age compared with women 25–29 years of age (Figure 1) and higher among obese women compared with those of normal BMI (Figure 2), after adjustment for other covariates. Prepregnancy and 3-month postpartum BMI were highly correlated (*r*_S_ = 0.9, *p* < 0.0001); consequently, associations between BMI and PBDE concentrations were the same regardless of whether we modeled prepregnancy or postpartum BMI or adjusted models for prenatal weight gain and percent body fat at 3 months postpartum. We observed no differences by parity or race (data not shown), but most women were Caucasian, limiting interpretation. Bearing in mind that all women in this study breast-fed at least 3 months in order to provide a sample, PBDE concentrations were not associated with breast-feeding duration (data not shown).

**Figure 3:**
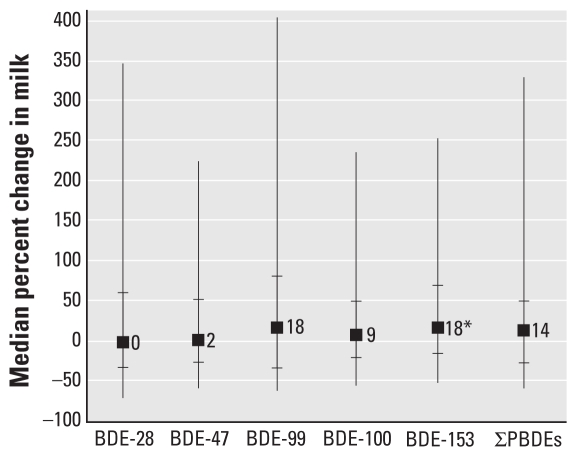
Percent change in PBDE concentration in milk between 3 and 12 months postpartum. Squares represent median; small horizontal bars represent the 25th and 75th percentiles; and vertical bars display the spread of the data between the 5th and 95th percentiles. *p < 0.05 for the sign test, testing the null hypothesis that the median percent change equals zero.

**Figure 4:**
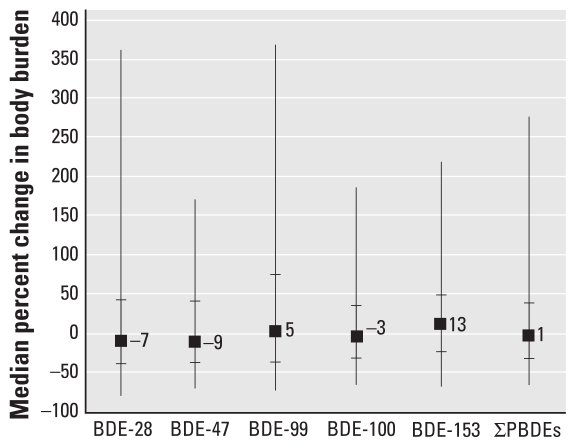
Percent change in estimated maternal PBDE body burden between 3 and 12 months postpartum, calculated as concentration (ng/g lipid) × body weight (kg) × body fat percentage (μg). Squares represent median percent change; small horizontal bars represent the 25th and 75th percentiles; and vertical bars display the spread of the data between the 5th and 95th percentiles.

Among the 83 women with two samples, the PBDE concentrations were highly correlated between milk samples taken at 3 and 12 months (*r*_S_ = 0.7–0.8, *p* < 0.0001; data not shown). The median ∑PBDE concentration at 3 months was 47 ng/g lipid (range, 8–2,010 ng/g lipid). Between 3 and 12 months, the change in ∑PBDE milk concentration ranged from −88% to 536%, but the median change (14%; interquartile range, −26% to 50%) was not significantly different from zero (*p* = 0.2). The magnitude and direction of change for most congeners varied considerably among individuals (Figure 3), but for BDE-153, the concentration significantly increased from 3 to 12 months postpartum (median change, 18%; interquartile range, −15% to 70%; *p* = 0.005). Considering women’s percent body fat along with their milk concentration, we estimated women’s median ∑PBDE body burden at 3 months to be 1,002 μg (interquartile range, 494–2,090 μg). The magnitude and direction of change in estimated body burden between 3 and 12 months varied among women (Figure 4), but the median change was not significantly different from zero for any congener or for ∑PBDE. Between 3 and 12 months postpartum, most women’s percent body fat decreased (median change, −6%; interquartile range, −12% to 0.6%; *p* < 0.0001), but the change was not associated with change in PBDE concentrations (data not shown). The lipid content in milk did not change markedly between 3 and 12 months of lactation (median change, −18%; interquartile range, −54% to 40%; *p* = 0.4).

## Discussion

∑PBDE concentrations ranged from 1 to 2,010 ng/g lipid in this large study of North Carolina breast milk samples collected between 2004 and 2006. These concentrations are higher than milk concentrations reported by most similarly timed studies from the United States (Johnson-Restrepo et al. 2007; Schecter et al. 2003; She et al. 2007; Wu et al. 2007) and considerably higher than those in reports from abroad (reviewed by Erdogrul et al. 2004; Frederiksen et al. 2009; Johnson-Restrepo et al. 2007; Lind et al. 2003; Toms et al. 2007). Concentrations in milk samples from this study were also higher, comparing lipid-adjusted concentrations (nanograms per gram milk lipid), than were corresponding concentrations in blood samples provided by mostly African-American pregnant women in a Maryland study during the same time period (Herbstman et al. 2007). The higher concentrations reported in these North Carolina samples could have resulted from a slightly younger population, regional differences in regulation and use of PBDEs across the United States, or simply a greater range revealed by the larger study size. Differences are not likely to be due to dramatic temporal increases over such a short period of time.

As reported in other studies, BDE-47 was present in highest concentration, followed by BDEs 99, 100, and 153, which we detected in nearly all samples. We detected BDEs 66, 85, 154, and 183 in < 70% of the samples. In this study, ΣPBDE included only congeners present in > 70% of the samples. This method is somewhat conservative and may underestimate total exposure, but minimizes assumptions inherent when summing data below the LOD. PBDE concentrations were not correlated with PCB or DDE concentrations, which supports reports suggesting that sources of exposure for these persistent pollutants are different (Frederiksen et al. 2009; Herbstman et al. 2007; Kiviranta et al. 2004; Schecter et al. 2005).

PBDE concentrations in milk varied by age and BMI but were not affected by parity or race. We had limited power to detect variability by race because the study population was predominantly white. We also lacked information needed to evaluate the effect of breast-feeding history among parous women, although it seems likely that most women breast-feeding their second child also breast-fed their first. When adjusted for other covariates, we observed the lower milk concentration among the oldest women compared with those 25–29 years of age for all congeners except BDE-28. Milk concentrations were significantly higher among obese women compared with those of normal weight for BDEs 47, 99, and 100, but were also slightly higher in milk from women with low BMI. The variability by weight and age, and across congeners, is similar to observations of prenatal serum samples from Maryland women (Herbstman et al. 2007) and general-population serum samples from the 2003–2004 National Health and Nutrition Examination Survey (Sjodin et al. 2008b). Higher concentrations among women in younger age groups may suggest that the environment and habits of younger generations expose them to higher levels of PBDEs than do those of women ≥ 35 years of age. Higher concentrations among heavier women may reflect the lipophilic nature of the chemicals. The slightly different pattern for BDE-153 may reflect differences in exposure sources and/or metabolism compared with other congeners.

We assessed variability by individual characteristics only among the congeners detected in > 70% of the participants. We were unable to measure BDE-209, the primary congener of the decaBDE formulation, which is the only brominated flame retardant still produced in the United States. BDE-209 is stable but less likely to bioaccumulate and be detected at remarkable levels in human tissue compared with the lower brominated congeners because of its short half-life (i.e., 2 weeks in humans) (Sjodin et al. 2003; Thuresson et al. 2006). According to the Agency for Toxic Substances and Disease Registry (2004), “the lower brominated PBDEs are much more likely than decaBDE to be stored in the mother’s body and released during pregnancy, cross the placenta, and enter fetal tissues. Because lower brominated PBDEs dissolve readily in fat, they can accumulate in breast milk fat and be transferred to babies and young children.”

For infants, the primary source of exposure to PBDEs is breast milk. Darnerud et al. (2001) estimated that infant exposure in Sweden in the late 1990s through breast milk averaged 0.11 μg/day for ΣPBDE, based on Swedish data assuming 3.7% fat in milk and 0.7 L/day consumption. The actual volume of milk consumed by the children in our study has not been determined; however, if we apply the same algorithm and substitute the median concentration observed in the 3-month samples from North Carolina, median daily exposure to infants through breast milk would be considerably higher: 0.8 μg BDE-47 and 1.5 μg ΣPBDEs.

Among women with samples at 3 and 12 months postpartum, the concentrations in milk were highly correlated and, for most congeners, did not significantly change. The range of change among all congeners was very large and tended to be in a positive (increasing) direction. Our results from this large sample generally differ from those of a small case series studying depuration, which reported subtle decreases in concentration over the course of lactation among eight women (Hooper et al. 2007). For BDE-153, however, the median concentration in our data significantly increased between 3 and 12 months postpartum. It is unclear why BDE-153 increased whereas other congeners did not. It may reflect variability in the distribution and skew of the change among congeners, which can affect the statistical power to detect change. It could also reflect different levels of continuous exposure over the postpartum period among the congeners. However, it is also important to consider possible variability in the metabolism and storage of the congeners. At 3 months postpartum, retention of excess fat and fluid from pregnancy may dilute the circulating chemical concentration and the corresponding concentration in milk samples. By 12 months, women have less pregnancy-associated fat and fluid retention, so some chemicals mobilized from fat stores could become more concentrated in milk. Differences in the level of change among congeners over the course of lactation may reflect variability in the metabolism, storage, and release of particular congeners from fat. Studies of other pollutants have suggested that weight loss increases chemical concentrations measured in fat tissue (Chevrier et al. 2000; Pelletier et al. 2003). In this cohort, neither maternal weight gain during pregnancy nor percentage of body fat was associated with PBDE concentration in milk. Furthermore, changes in the woman’s percentage of body fat postpartum were not associated with changes in milk PBDE concentration.

All women in this study breast-fed at least 3 months, which was required to enter the study. Among these women, PBDE concentration was not associated with breast-feeding duration through 12 months postpartum. However, we were unable to assess whether exposure to PBDEs could impair a woman’s ability to initiate breast-feeding or decrease milk supply enough cause her to discontinue breast-feeding before 3 months postpartum, when women entered our study.

This is the largest study of breast milk PBDE concentration in the United States to date and the only one that evaluated differences by individual characteristics such as age, BMI, parity, and duration of breast-feeding. However, this study was limited to mostly Caucasian, well-educated mothers from central North Carolina. Other U.S. studies of PBDE concentrations in breast milk also included predominantly white and well-educated populations (She et al. 2007; Wu et al. 2007), which is reflective of breast-feeding women in the United States (van Rossem et al. 2009). Although there is no reason to suspect that this North Carolina population would systematically differ from others, the data should be interpreted as regional and not representative of the United States.

The persistence of PBDEs raises our vigilance for potential adverse environmental and human health consequences. Similar persistent organic pollutants such as *p*,*p′*-DDE and PCBs have been extensively investigated over the past 20–30 years for reproductive and developmental toxicity (Branchi et al. 2003; Tilson and Kodavanti 1997, 1998; Tilson et al. 1990, 1998), resulting in restrictions on many compounds in the United States (Cordle et al. 1982). Although levels of these other organic pollutants are steadily decreasing, levels of PBDEs are still elevated in the United States compared with other countries with limited recent use.

The consequences of exposure to PBDEs are unknown. Rodent studies have suggested that perinatal exposure to PBDEs may cause learning delays and behavioral problems that persist into adulthood, possibly worsening with age (Eriksson et al. 2001a, 2001b, 2002; Rice et al. 2007; Viberg et al. 2002, 2003a, 2003b, 2004). Most rodent studies have focused on the pentaBDE congeners, exposing mice during gestation and soon after birth to various doses.

To date, little information is available to assess human health effects. Given the extensive nutritional and immunologic benefits of breast milk, promotion of breast-feeding as the best method for infant feeding should continue (Oddy 2001; Schack-Nielsen and Michaelsen 2007). However, timely studies are needed to continue to monitor levels, to evaluate health effects, and to minimize exposure to all pervasive, persistent pollutants (Hooper and She 2003).

Ideally, as PBDE containing products exit the market, levels will begin to decline in the United States, as they have in Europe and other areas where use ended in the late 1990s (Lind et al. 2003). However, because household products containing PBDEs will continue to be in use for some time, it is important to observe generations with greater exposure and monitor whether adverse health outcomes are associated with high-level exposures, especially among the most vulnerable populations. Human milk samples offer a minimally invasive opportunity to monitor population level trends in PBDE exposure in the coming years (Hooper and She 2003; Meironyte et al. 1999; Wu et al. 2009).

## Figures and Tables

**Table 1:** Summary of studies^a^ reporting ∑PBDE concentrations in human milk samples from the United States.

	Year of sample (no.)				
Characteristic	2002 (47)	2003 (40)	2004 (38)	2004 (46)	2004–2006 (301)
PBDE concentration					
Mean ± SD	74 ± 103	96	76 ± 304	NA	89 ± 146.7
Median	34	50	20	30	51
Range (ng/g lipid)	6–419	6–321	0.1–1910	4–264	1–2,010
Mean percent lipid	3	4	2 ± 2	NA	2
Mother’s age (mean years ± SD)	29 ± 6	31 ± 6	35 ± 5	NA	31 ± 5
Timing of collection (weeks postpartum)	NA	2–8	NA	2–8	12–24
Location	Austin, TX	Pacific Northwest	Central MA	Boston, MA	Central NC
Reference	Schecter et al. 2003	She et al. 2007	Johnson-Restrepo et al. 2007	Wu et al. 2007	Present results

NA, not available from published manuscript.

a Statistics here differ slightly from those originally published because of rounding. The congeners comprising ∑PBDE may differ slightly among studies but generally include the congeners included in the present study: BDE-28, −47, −99, −100, and −153.

**Table 2:** Lipid-adjusted concentrations of brominated flame retardants (ng/g lipid) in 303 human milk samples collected around 3 months postpartum from central North Carolina, 2004–2006.

PBDE congener	IUPAC number	Detectable [no. (%)]	Minimum	25th percentile	Median	75th percentile	Maximum
2,4,4-Tri-BDE	28	295 (97)	ND	1	2	4	50
2,2′,4,4′-Tetra-BDE	47	302 (99)	ND	16	28	54	1,430
2,3′,4,4′-Tetra-BDE	66	139 (46)	ND	ND	ND	0.5	11
2,2′,3,4,4′-Penta-BDE	85	196 (65)	ND	ND	0.5	1	27
2,2′,4,4′,5-Penta-BDE	99	278 (92)	ND	3	5	11	299
2,2′,4,4′,6-Penta-BDE	100	300 (99)	ND	2	5	10	188
2,2′,4,4′,5,5′-Hexa-BDE	153	302 (99)	ND	3	6	14	229
2,3,4,4′,5,6-Hexa-BDE	154	163 (54)	ND	ND	0.3	0.7	13
2,2′,3,4,4′,5′,6-Hepta-BDE	183	35 (12)	ND	ND	ND	ND	15
∑PBDEs		303	1.2	28	51	102	2,010

ND (nondetected) values are treated as (median LOD/square root of 2) in the calculation of the mean.
